# Coronary Artery Tortuosity Found in a Marathon Runner

**DOI:** 10.7759/cureus.39849

**Published:** 2023-06-01

**Authors:** Humail Patel, Danielle Diem, Patrick Keyes, Azhar A Supariwala, Sarah Syed

**Affiliations:** 1 Internal Medicine, Northwell Health, Manhasset, USA; 2 Cardiology, Northwell Health, Bay Shore, USA

**Keywords:** vascular disease, rigorous exercise, marathon runner, anomalous coronaries, coronary artery tortuosity

## Abstract

Coronary artery tortuosity (CAT) is an anatomical anomaly in which the coronary arteries contain kinks or coils. It is usually found incidentally in elderly patients with long-standing uncontrolled hypertension. This case illustrates a 58-year-old female marathon runner who was found to have CAT, originally presenting with chest pain, hypotension, presyncope, and a severe cramping sensation in her legs.

## Introduction

It is commonly understood that regular low- to moderate-intensity physical exercise can aid in preventing cardiovascular disease and is associated with a decrease in all-cause mortality [1]. On the other hand, it has also been well established that high amounts of vigorous exercise can increase the risk for and, in some cases, even trigger adverse cardiovascular events such as sudden cardiac death or myocardial infarction, even in healthy young athletes [2,3]. In patient populations who regularly engage in high-intensity exercise such as marathon runners, standardized pre-participation cardiovascular screening and individualized risk stratification may be of benefit [4]. Among these patients, 19% of adverse cardiovascular events are due to coronary artery anomalies for those under the age of 35-40, while 80% of adverse cardiovascular events are due to coronary artery disease (CAD) for those who are over the age of 35-40 [4].

One commonly encountered anatomical anomaly is the presence of coronary artery tortuosity (CAT), which is considered a benign finding in most cases. However, more severe cases of CAT can lead to grave coronary issues such as myocardial ischemia [5]. Although the exact pathogenesis of coronary artery tortuosity is not known, it has been shown to, at least in part, result from aging, due to an overall reduction of the amount of elastin in the arterial wall [5], as well as long-standing hypertension [6]. One study demonstrated that patients with smaller left ventricular mass and a relatively greater left ventricular shortening along the longitudinal axis were more likely to have CAT, indicating that CAT may occur as an adaptive response to adequately distribute the exaggerated force onto the coronaries as a result of this dynamic shortening and deformation [7].

Coronary artery tortuosity was found to be prevalent in patients with spontaneous coronary artery dissection (SCAD), and severe tortuosity has been associated with recurrent SCAD events. There was also noted to be a higher prevalence of CAT among patients with takotsubo cardiomyopathy [6]. Another study of 32 patients revealed that all patients with fibromuscular dysplasia (FMD) also had CAT [8]. CAT may also be one manifestation of arterial tortuosity syndrome (ATS), which may predispose patients to catastrophic aneurysm formation of the aorta and several mid-sized arteries [9].

## Case presentation

**Figure 1:**
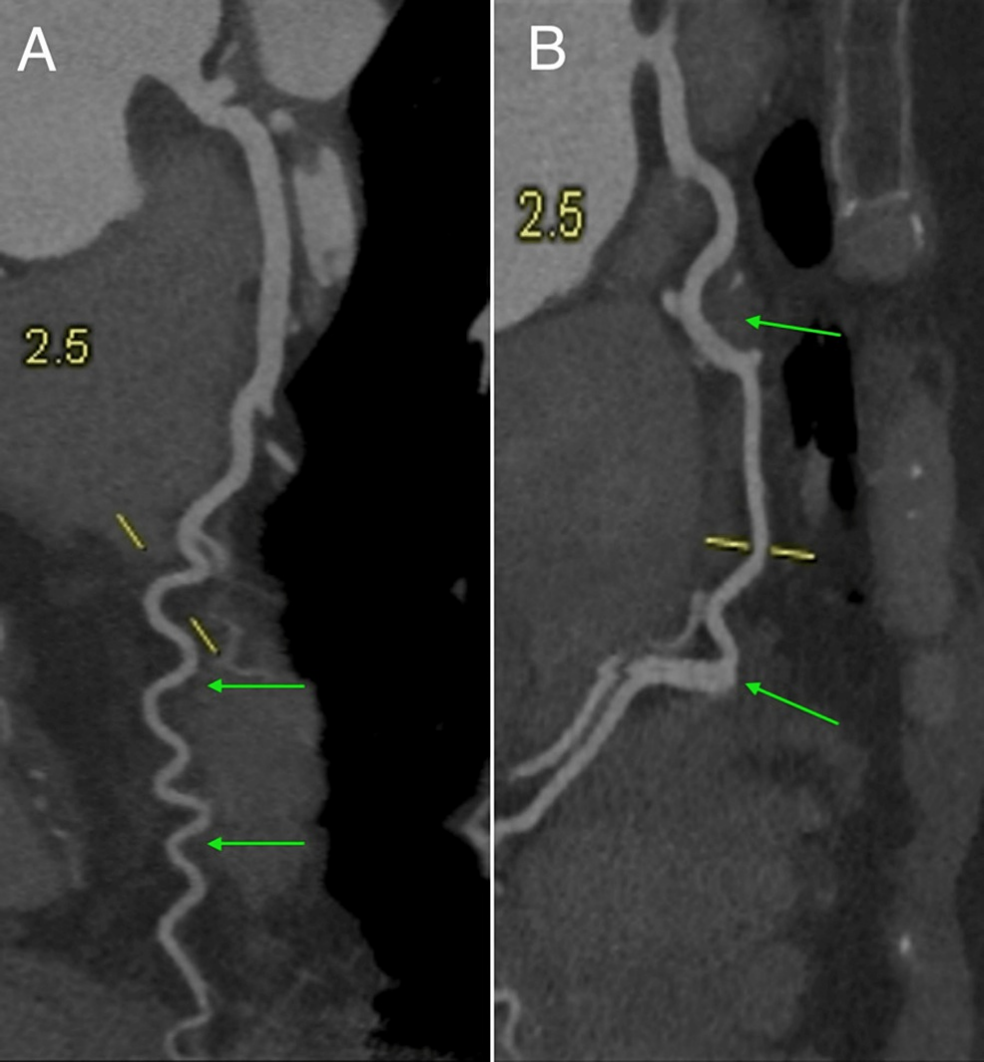
CTA showing (A) significant tortuosity of the left anterior descending and left circumflex arteries (green arrows) and (B) significant tortuosity of the posterior descending artery (green arrows) There were no regional wall motion abnormalities. The ejection fraction is 68%. CTA: computed tomography angiography

**Figure 2:**
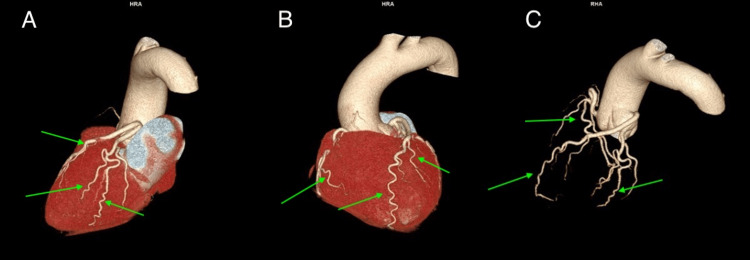
3D reconstruction of the coronary CTA (A) Significant tortuosity of multiple anterior coronary arteries (green arrows). (B) Significant tortuosity of multiple posterior coronary arteries (green arrows). (C) Heart removed, demonstrating the degree of tortuosity of several coronary arteries (green arrows). 3D: three dimensional, CTA: computed tomography angiography

A 58-year-old female marathon runner with a past medical history of dyslipidemia and mild mitral regurgitation presented to the cardiology clinic with complaints of chest pain, presyncope, and a cramping sensation in her legs. The patient reported severe pain in her bilateral lower extremities and a subjective drop in blood pressure while running. The patient’s most recent episode was one month prior to presentation after completing a half marathon. During that episode, she experienced leg cramping throughout the race that worsened upon completion. In addition, she reported becoming pale and hypotensive to 70/40 mmHg. The patient also reported a one-year history of arm, leg, and back cramping and a one-month history of chest discomfort. Upon presentation to the clinic, the patient’s vital signs were notable for a blood pressure of 108/68 mmHg and a heart rate of 56 beats per minute. Physical examination was unremarkable. Electrocardiogram (ECG) revealed sinus bradycardia with a rate of 47 beats per minute. The patient was given an event monitor and underwent evaluation with a computed tomography angiography (CTA), which revealed significant tortuosity of the left anterior descending, circumflex, and right coronary arteries, with no evidence of coronary artery disease (CAD), coronary calcifications, atherosclerosis, or identifiable stenosis (Figure 1). Three-dimensional (3D) reconstruction of the coronaries revealed the extent of the tortuosity (Figure 2).

The patient was noted to have normal left ventricle size and function. The patient was instructed to closely monitor her symptoms and use the event monitoring device until her follow-up appointment. The patient’s event monitor displayed consistent sinus rhythm, with some tachycardic and bradycardic events. The patient continued to have intermittent palpitations described as fluttering sensations every few days that lasted for seconds at a time. These episodes were sometimes associated with lightheadedness, chest tightness, and arm pain. The patient also reported feeling fatigued and noted that as a result, she had decreased her amount of exercise significantly. As per her event monitor, premature ventricular contractions were noted in association with the reported symptoms of lightheadedness and chest pain. Additional events included three episodes of paroxysmal supraventricular tachycardia and some atrial tachycardia. For hypotension regulation, the patient was instructed to be liberal with salt intake and wear compression stockings. For the cramping, the patient was advised to take magnesium and coenzyme Q10 supplements and was counseled on sufficient water intake. The patient denied any syncopal or presyncopal episodes since her initial visit.

## Discussion

Although CAT in and of itself may be a benign condition, its association with other more severe vascular diseases such as spontaneous coronary artery dissection (SCAD), fibromuscular dysplasia (FMD), or arterial tortuosity syndrome (ATS) may provide some value in the early detection or screening of these conditions. If identified early, treatment can be initiated, and interventions can be offered to prevent and delay late-stage and often catastrophic complications.

More research is needed to determine if there is any association between high amounts of vigorous exercise and the development of CAT as seen in our patient. This may be possibly due to excessive strain secondary to increased myocardial oxygen demand in the setting of underlying genetic susceptibility. Further studies are also needed to outline the pathophysiology and potential clinical complications of CAT, especially in cases such as this one, where there is no other definitive explanation for the patient’s underlying anatomy or presenting symptomatology. Geometric analyses of coronary anatomy and plaque locations have revealed that sharp angulations in the coronaries may cause symptoms of ischemia due to altered coronary hemodynamic flow and varying degrees of endocardial shear stress [10]. Therefore, although it cannot be definitively linked, the patient’s anomalous coronary anatomy may have contributed to her clinical picture.

In athletes or people who engage in high amounts of vigorous aerobic exercise, assessing the coronary anatomy via a CTA may be warranted and may play a role in pre-participation risk stratification to prevent future unexpected cardiac events. If this coronary anomaly is identified, especially in the absence of older age or chronic hypertension, further testing should be considered to potentially identify an associated and likely more harmful disease process.

## Conclusions

Coronary artery tortuosity is most seen in elderly patients with long-standing hypertension. This case presents a unique finding of tortuous coronary arteries in an otherwise healthy female marathon runner in the absence of any major cardiac risk factors. Although the patient’s symptoms cannot be definitively linked to CAT, its association with other more severe conditions may shed light on its underlying pathophysiology and its role as a diagnostic tool.
